# YBX1 promotes tumor growth by elevating glycolysis in human bladder cancer

**DOI:** 10.18632/oncotarget.19583

**Published:** 2017-07-26

**Authors:** Liuyu Xu, Hongyun Li, Longchao Wu, Shiming Huang

**Affiliations:** ^1^ Department of Urology, QianFoShan Hospital Affiliated to Shandong University, Jinan 250014, P. R. China; ^2^ Department of Urology, Penglai People’s Hospital of Shandong, Penglai 265600, P. R. China

**Keywords:** YB-1, Warburg effect, The Cancer Genome Atlas, bladder cancer

## Abstract

Aerobic glycolysis, also known as Warburg effect, is a key hallmark of cancers. The Y-box-binding protein 1 (YBX1) is a well-known oncoprotein implicated in multiple malignant phenotypes of cancers. Meanwhile, little is known about the oncogenic functions and mechanisms of YBX1 in bladder cancer. Based on gene set enrichment analysis (GSEA) of TCGA RNAseq data, we find that YBX1 was profoundly involved in the glycolysis part of glucose metabolism. Loss- and gain-of-function studies show that YBX1 can enhance glycolysis as revealed by expression of glycolytic enzymes, glucose uptake, lactate secretion and extracellular acidification rate (ECAR). Inhibition of glycolysis completely compromises the tumor-promoting effect of YBX1 on tumor growth. Mechanistically, YBX1 regulates the expression of c-Myc and HIF1α, which further upregulate glycolytic enzymes to facilitate glycolysis. Moreover, *in vivo* study further confirms that genetic silencing of YBX1 markedly attenuates tumor growth and this tumor-suppressive effect is largely dependent on reduced glycolysis. Taken together, these results, as a proof of principle, provide a novel insight into the oncogenic role of YBX1 in glycolysis and suggest the potential therapeutic strategy by targeting YBX1 in bladder cancer.

## INTRODUCTION

Bladder cancer, a typical malignant urogenital tract cancer, is the fifth and fourth most common malignancy in Europe and the USA, respectively [1, 2]. It affects about 300,000 people each year worldwide. The disease can be categorized into two groups: superficial bladder cancer and muscle-invasive bladder cancer. Although radical cystectomy, pelviclymphadenectomy, and neoadjuvant chemotherapy are available to the treatment of bladder cancer, the prognosis is still poor due to its high prevalence and recurrent nature [2]. Therefore, it is of paramount importance to underlying the molecular mechanisms of bladder cancer progression and to develop specific therapeutic targets for management of bladder cancer.

A distinctive character of cancer cells is their altered carbohydrate metabolism [3]. Cancer cells convert glucose to lactate instead of oxidative phosphorylation even in the presence of oxygen, also known as aerobic glycolysis or Warburg effect [4]. Because it is highly associated with the malignant phenotypes of cancer, aerobic glycolysis is considered a metabolic signature for invasive cancer, therapeutic targeting of glycolysis in cancer patients has been an attractive proposition [5]. The glycolytic phenotype confers cancer cells with several selective advantages. On the one hand, enhanced glycolysis ensures ATP levels compatible with the demands of fast proliferating tumor cells. On the other hand, glycolysis provides a constant supply of metabolic intermediates, which are essential for macromolecule biosynthesis and rapid cell proliferation [6, 7].

Y-box binding protein 1 (YBX1), is a member of the cold-shock protein superfamily that binds both DNA and RNA to orchestrate transcription and translation, pre-mRNA splicing, DNA repair, and mRNA packaging [8, 9]. Recently, emerging studies have indicated that dysregulation of YBX1 is linked to tumor progression, including prostate cancer [10], breast cancer [11], and bladder cancer [12]. YBX1 plays diverse pro-oncogenic roles in cancers, such as malignant growth, invasion, metastasis, chemotherapy resistance and tumor angiogenesis [13]. Meanwhile, YBX1 is regarded as a poor prognostic factor in breast cancer [14], ovarian cancer [15], and gastric cancer [16]. In bladder cancer, patients with high YBX1 expression had lower overall survival rates [12]. And YBX1 is involved in cell growth, invasion, motility and resistance to cisplatin and doxorubicin [17]. However, limited knowledge is known about the underlying mechanism by which YBX1 promotes tumor progression.

In this study, we showed that YBX1 could promote glycolysis in bladder cancer cells by modulating Myc and HIF1α expression, which further facilitate the expression of glycolytic enzymes and ultimately contribute to tumor progression.

## RESULTS

### YBX1 is involved in the glycolytic phenotype of bladder cancer cells

**Figure 1:**
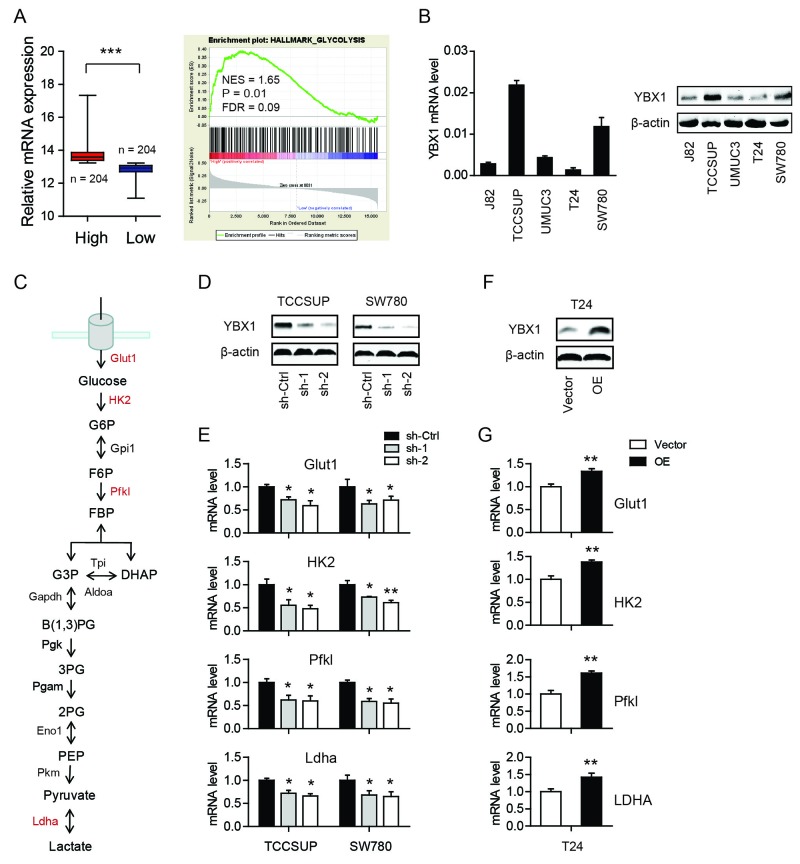
YBX1 is involved in the glycolytic phenotype of bladder cancer cells **(A)** GSEA plot of glycolysis pathway based on the high-YBX1 versus low-YBX1 expression profiles. NES: normalized enrichment score; FDR: false discovery rate. **(B)** The mRNA and protein level of YBX1 in bladder cancer cells. **(C)** Summary of the glycolytic enzymes and metabolites. Glycolytic enzymes detected were highlighted in red. **(D)** The knockdown efficiency of YBX1 in TCCSUP and SW780 cells. **(E)** The effect of YBX1 knockdown on the mRNA expression of Glut1, HK2, Pfkl and Ldha. **(F)** The overexpression efficiency of YBX1 in T24 cells. **(G)** The effect of YBX1 overexpression on the mRNA expression of Glut1, HK2, Pfkl and Ldha. *, P < 0.05, **, P < 0.01.

**Figure 2:**
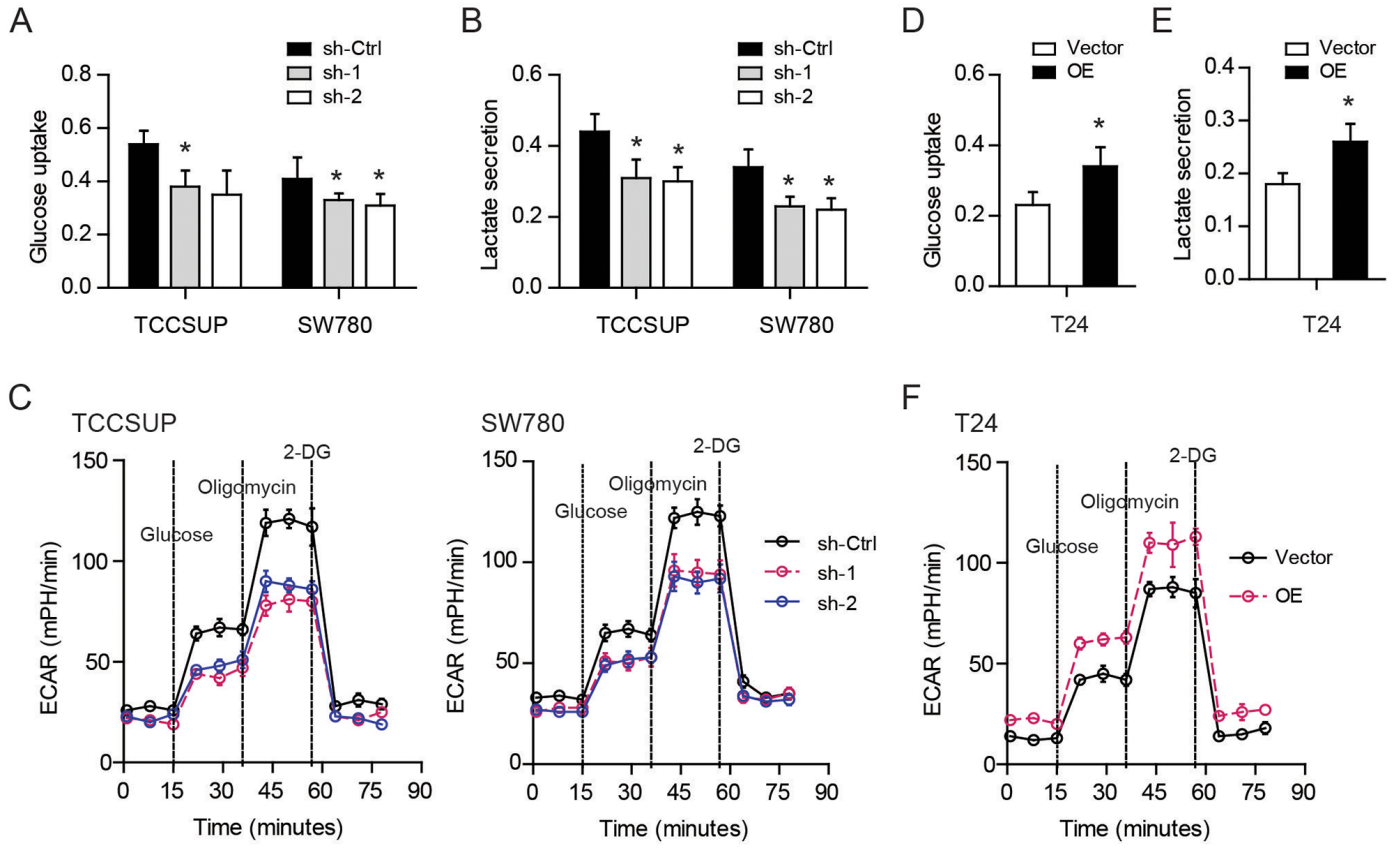
YBX1 promotes glycolysis in bladder cancer cells **(A-C)** The effect of YBX1 knockdown on the glucose uptake (A), lactate secretion (B), and extracellular acidification ratio (C) of TCCSUP and SW780 cells. **(D-F)** The effect of YBX1 overexpression on the glucose uptake (D), lactate secretion (E), and extracellular acidification ratio (F) of T24 cells. *, P < 0.05.

To identify the cellular functions of YBX1 in bladder cancer, we performed gene set enrichment analysis (GSEA) in TCGA cohort. Based on the expression level of YBX1, the TCGA cohort was divided into two groups: high-YBX1 group and low-YBX1 group (Figure 1A). GSEA results showed that genes involved in glycolysis pathway were particularly enriched in the high-YBX1 group, suggesting the potential regulatory roles of YBX1 in glucose metabolism (Figure 1A). The reprogramming of glucose to support continuous proliferation is a hallmark of cancer. To test whether YBX1 is able to modulate glucose metabolism, loss-of-function and gain-of-function studies were carried out. The mRNA and protein level of YBX1 in five bladder cancer cell lines was measured. Notably, TCCSUP and SW780 cells had higher YBX1 level, while T24 had the lowest (Figure 1B). Therefore, we silenced YBX1 in TCCSUP and SW780 cells and overexpressed YBX1 in T24 cells. Two sh-RNAs targeting YBX1 both led to pronounced decrease in YBX1 protein level in TCCSUP and SW780 cells (Figure 1D), whereas overexpression of YBX1 resulted in near to 4-fold increase of YBX1 level in T24 cells (Figure 1F). There are several key enzymes involved in glycolysis, including glucose transporter 1 (Glut1), hexokinase 2 (HK2), phosphofructokinase, liver type (Pfkl), and lactate dehydrogenase A (Ldha) (Figure 1C). We found that knockdown of YBX1 significantly inhibited the expression of these glycolytic enzymes in TCCSUP and SW780 cells (Figure 1E). On the contrary, overexpression of YBX1 in T24 cells drastically increased the mRNA level of glycolytic genes (Figure 1G). Consistently, knockdown of YBX1 also resulted in a decrease in glucose uptake (Figure 2A) and lactate secretion (Figure 2B) in TCCSUP and SW780 cells. In T24 cells, overexpression of YBX1 enhanced glucose consumption (Figure 2D) and lactate production (Figure 2E). Furthermore, to further determine the glycolytic phenotype, YBX1 knockdown or overexpression cells were subjected to Seahorse Extracellular Flux analysis to assess the cellular bioenergetic activity. The results showed that YBX1 loss significantly decreased the extracellular acidification rate (ECAR) associated with glycolytic capacity in TCCSUP and SW780 cells (Figure 2C). Meanwhile, overexpression of YBX1 significantly increased ECAR associated with glycolytic capacity of T24 cells (Figure 2F). Taken together, with results obtained from YBX1 knockdown and overexpression cells, we demonstrate that YBX1 plays an important role in regulating glycolysis in bladder cancer.

### YBX1 promotes tumor growth by enhancing glycolysis in bladder cancer

**Figure 3:**
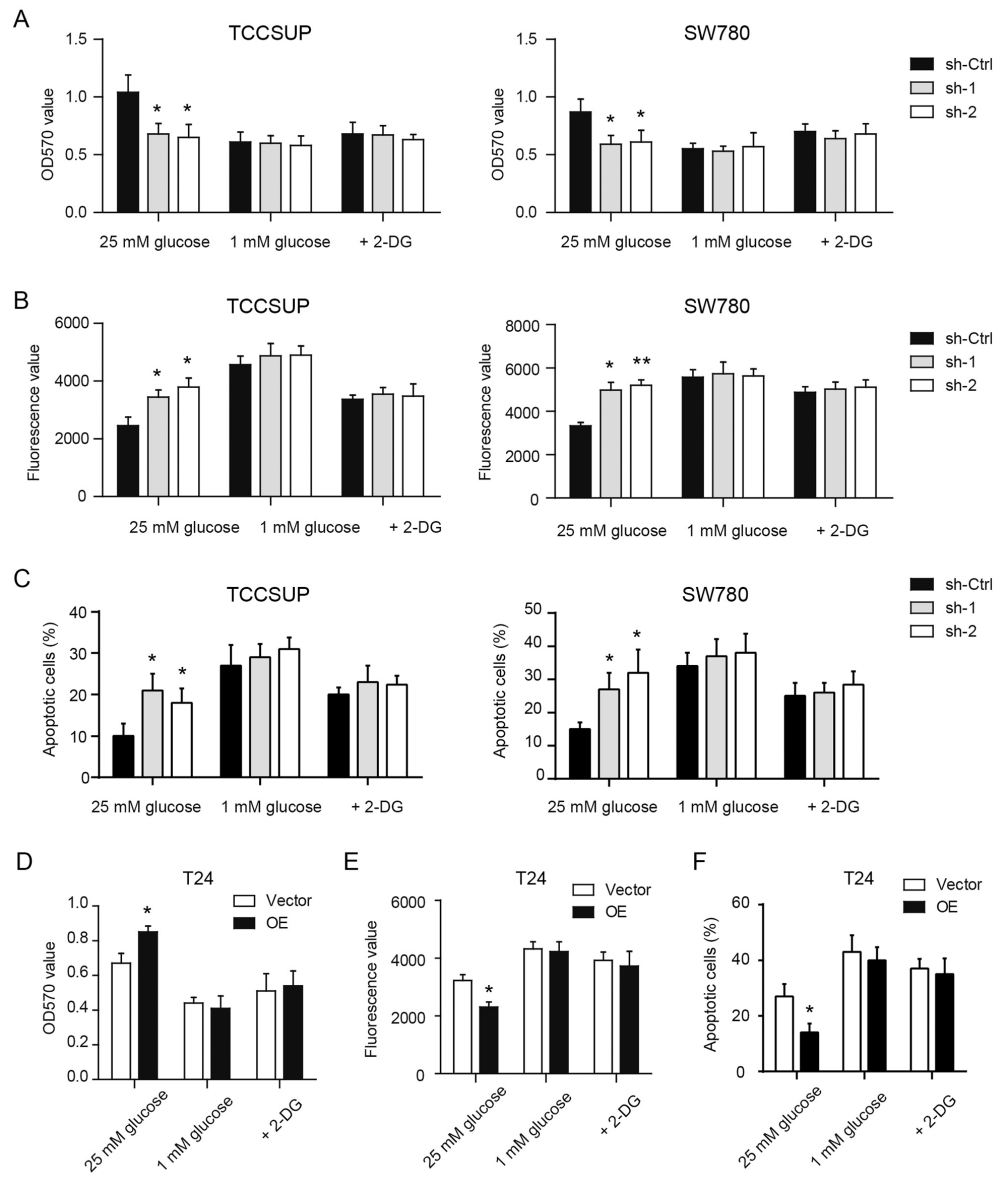
YBX1 promotes tumor growth by enhancing glycolysis of bladder cancer In the presence of high glucose medium (25mM), low glucose medium (1 mM) and 2-DG (20 mM), MTT assay and caspase-3/7 activity assay were used to measure cell viability and apoptosis in YBX1 knockdown and overexpression cells. **(A-C)** The effect of YBX1 knockdown on the cell viability (A) and apoptosis (B, C) of TCCSUP and SW780 cells with indicated treatment. **(D-F)** The effect of YBX1 overexpression on the cell viability (D) and apoptosis (E, F) of T24 cells with indicated treatment. For caspase-3/7 activity assay, a spectrofluorometer was used to detect caspase-3/7 activity at an excitation wavelength range of 485 ± 20 nm and an emission wavelength range of 530 ± 25 nm. Fluorescence value of each well was recorded and normalized to the total protein. *, P < 0.05, **, P < 0.01.

Next, to test potential oncogenic functions of YBX1 in bladder cell, we firstly determined cell proliferation and apoptosis in stable control and YBX1 knockdown cells cultured in regular growth medium. As shown in Figure 3A, YBX1 loss resulted in decreased cell viability of TCCSUP and SW780 cells after 3 days culture. In serum-starvation induced cell apoptosis, silencing of YBX1 significantly promoted cell apoptosis of TCCSUP and SW780 cells as revealed by increased caspase-3/7 activity (Figure 3B) and Annexin V/PI staining assay (Figure 3C). To test whether the effects of YBX1 on cell proliferation and apoptosis are glycolysis-dependent, we altered the glucose concentration in the culture medium to 1 mM to limit glucose utilization. Meanwhile, 2-DG, a famous glycolysis inhibitor, was used to block glycolysis. We found that both low glucose level and 2-DG can sufficiently abolish the implication of YBX1 knockdown on cell proliferation (Figure 3A) and apoptosis (Figure 3B, 3C) of TCCSUP and SW780 cells. In T24 cells, overexpression of YBX1 exhibited prolific (Figure 3D) and anti-apoptotic roles (Figure 3E, 3F); and similarly, inhibition of glycolysis by glucose remove or 2-DG completely compromised the oncogenic functions of YBX1 (Figure 3D-3F). Collectively, these data indicate that increased glucose utilization may contribute to the malignant potential of YBX1 in bladder cancer.

### YBX1 regulates Myc and HIF1α expression in bladder cancer cells

**Figure 4:**
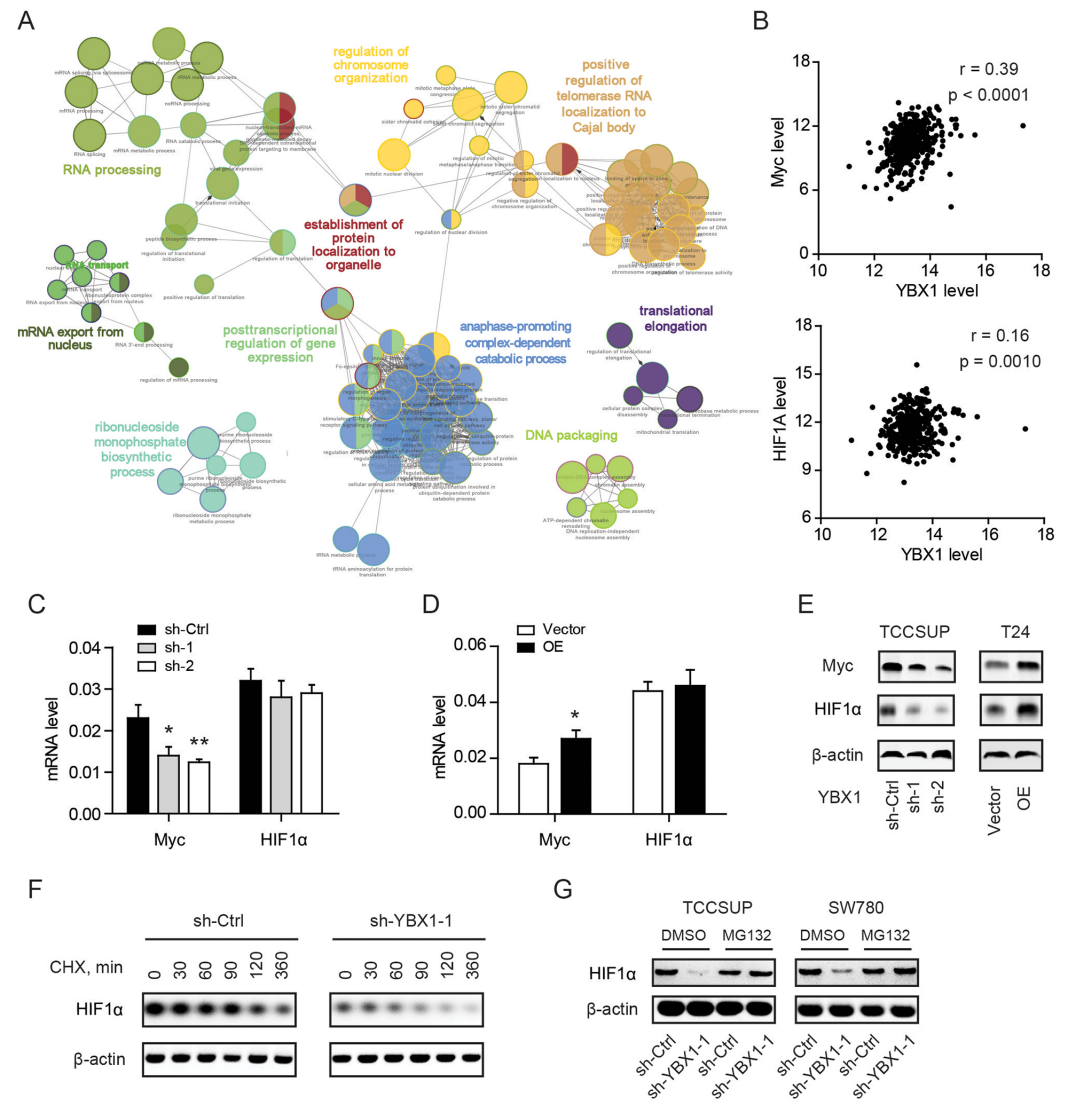
YBX1 regulates Myc and HIF1α expression in bladder cancer cells **(A)** ClueGO + CluePedia analysis of enriched pathways on the top 300 YBX1 positively correlated genes by CytoScape. Nodes represent enriched pathways grouped based on shared genes and linked based on κ score. **(B)** The correlation between YBX1 and Myc and HIF1α was analyzed by TCGA RNAseq data (n = 408). **(C)** and **(D)** The effect of YBX1 knockdown (C) and overexpression (D) on the mRNA level of Myc and HIF1α in corresponding cells. (E) The effect of YBX1 knockdown and overexpression on the protein level of Myc and HIF1α in indicated cells. **(E-F)** Effect of cycloheximide (CHX) or MG132 on the expression of HIF1α protein in sh-Ctrl and sh-YBX1 TCCSUP cells. TCCSUP cells were initially exposed to cycloheximide (E) or MG132 (F) for the indicated times. At each time, whole cell lysates were prepared and analyzed by Western blotting analysis. *, P < 0.05, **, P < 0.01.

YBX1 is a versatile molecule that possesses multiple biological functions in both the nucleus and cytoplasm [18]. To evaluate the biological processes mediated by YBX1 in bladder cancer, we annotated the top 300 YBX1 positively correlated genes by using TCGA RNAseq data (https://hgserver1.amc.nl/cgi-bin/r2/main.cgi). ClueGO and CluePedia analysis showed that the correlated genes were associated with RNA processing, regulation of chromosome organization, mRNA export from nucleus, posttranscriptional regulation of gene expression, anaphase-promoting complex-dependent catabolic process, translational elongation, and etc (Figure 4A). All these processes pointed to the regulatory role of YBX1 in a DNA/RNA-dependent manner. Myc and HIF1α are two crucial regulators in glycolysis and certainly contribute to the glycolytic phenotype of bladder cancer [6, 19]. We therefore hypothesized whether YBX1 is able to regulate Myc and HIF1α to promote glycolysis. Data derived from TCGA showed that YBX1 was closely correlated with the Myc and had a meager correlation with HIF1α (Figure 4B). YBX1 loss led to marked decrease of Myc but not HIF1α at mRNA level of TCCSUP cells (Figure 4C). And reversely, overexpression of YBX1 increased Myc but not HIF1α at mRNA level of T24 cells (Figure 4D). However, knockdown of YBX1 reduced both Myc and HIF1α at protein level, and overexpression of YBX1 increased Myc and HIF1α protein (Figure 4E). Subsequently, we investigated the time course for the effect of YBX1 knockdown on HIF1α expression. In the presence of 40 μg/ml CHX, a translational inhibitor, we found that HIF1α protein degraded more quickly in YBX1 knockdown cells (Figure 4F). To further test whether the regulation of YBX1 on HIF1α protein is the ubiquitin-proteasome pathway dependent, we treated sh-Ctrl and sh-YBX1 cells with MG132 (4 μM), a 26S proteasomal inhibitor. As a result, HIF1α degradation in sh-YBX1 cells was largely abrogated by proteasome inhibition (Figure 4G). This data suggesting YBX1 can modulate Myc and HIF1α expression through different mechanism.

### Myc and HIF1α link the YBX1-mediated glycolytic phenotype of bladder cancer cells

**Figure 5:**
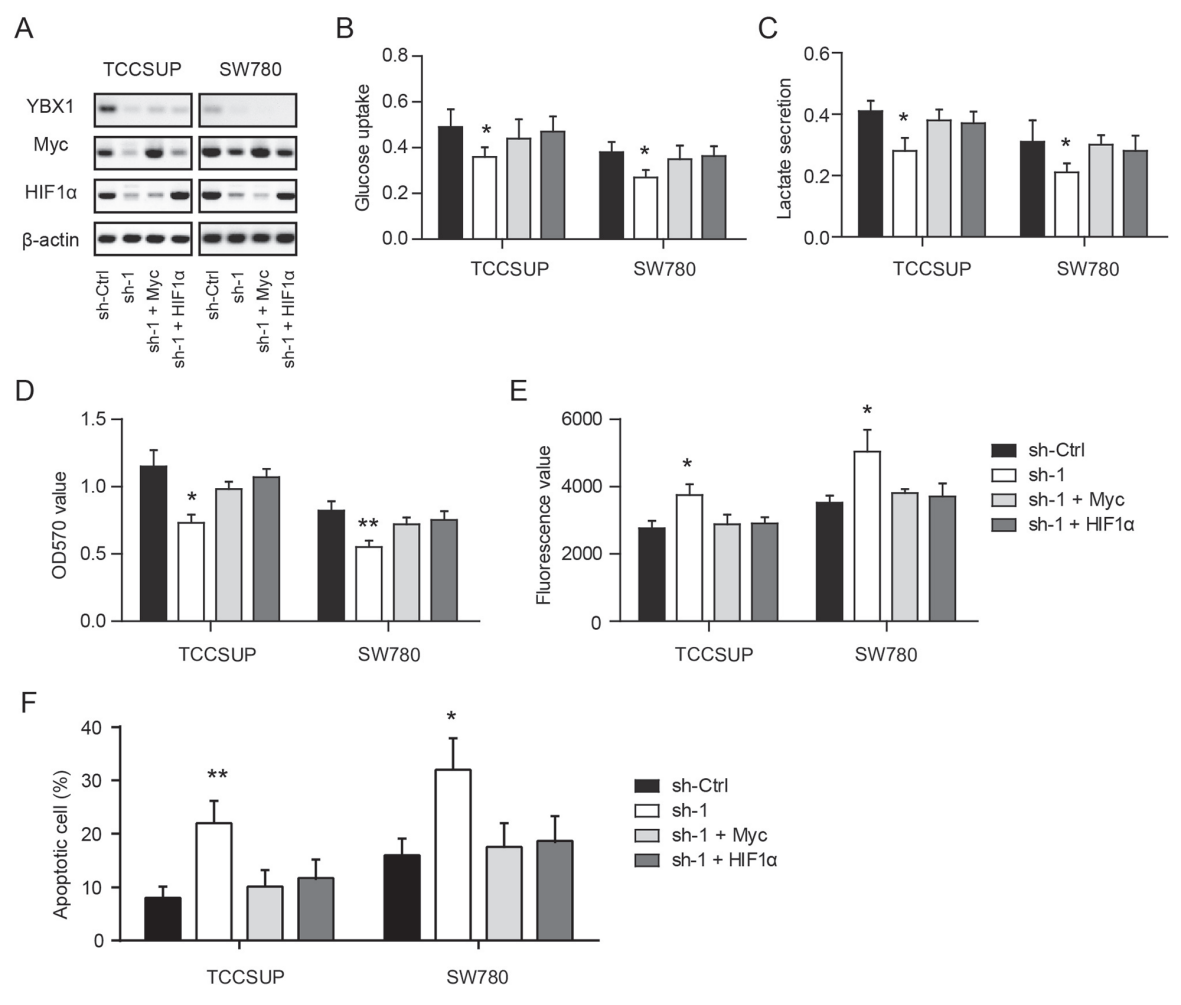
Myc and HIF1α link the YBX1-mediated glycolytic phenotype of bladder cancer cells **(A)** The protein level of Myc and HIF1α in TCCSUP and SW780 cells were measured in the presence of YBX1 knockdown and Myc or HIF1α overexpression. **(B-F)** Effects of YBX1 knockdown and Myc or HIF1α overexpression on the glucose uptake (B), lactate secretion (C), cell viability (D) and cell apoptosis (E, F) in TCCSUP and SW780 cells. *, P < 0.05, **, P < 0.01.

To test whether Myc or HIF1α mediates the YBX1-induced glycolytic phenotype and cellular functions in bladder cancer, we introduced Myc and HIF1α to YBX1 knockdown cells. As shown in Figure 5A, YBX1 loss induced reduction of Myc and HIF1α can be recovered by their respective plasmids. And interestingly, either introduction of Myc or HIF1α can completely abolish the inhibitory effects of YBX1 knockdown on glycolysis, as demonstrated by glucose uptake (Figure 5B) and lactate secretion (Figure 5C). Meanwhile, YBX1-induced growth inhibition (Figure 5D) and apoptosis (Figure 5E, 5F) can also be recovered by overexpression of Myc or HIF1α. Collectively, these results suggest that YBX1-mediated glycolytic activities and oncogenic functions are largely dependent on its regulatory roles on Myc and HIF1α expression.

### Genetic silencing of YBX1 inhibits tumor growth *in vivo*

**Figure 6:**
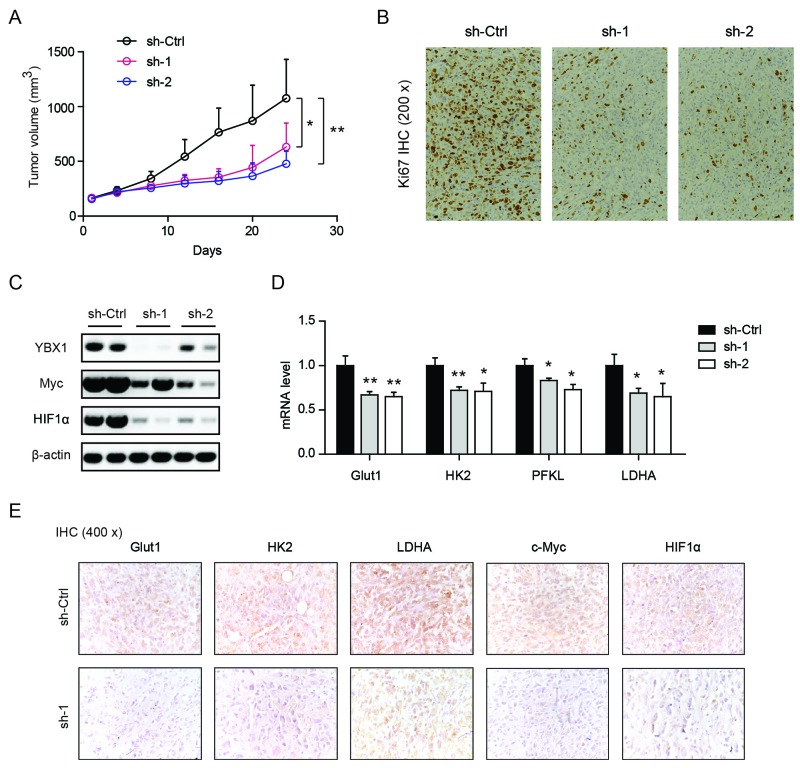
Genetic silencing of YBX1 inhibits tumor growth *in vivo* **(A)** Growth curves of subcutaneous xenograft from sh-Ctrl, sh-YBX1-1 (sh-1) and sh-YBX1-2 (sh-2) cells. **(B)** Immunohistochemical analysis of Ki67 staining among sh-Ctrl, sh-1 and sh-2 groups. **(C)** Western blotting analysis of YBX1, Myc and HIF1α expression among sh-Ctrl, sh-1 and sh-2 groups. **(D)** Real-time qPCR analysis of Glut1, HK2, Pfkl and Ldha expression among sh-Ctrl, sh-1 and sh-2 groups. **(E)** IHC analysis of Glut1, HK2, Ldha, Myc, and HIF1α protein expression in sh-Ctrl and sh-1 groups. *, P < 0.05, **, P < 0.01.

To further determine the oncogenic role of YBX1 *in vivo*, a subcutaneous xenograft model was used. As shown in Figure 6A, tumors derived from two-shRNAs targeting YBX1 grew much slower than sh-Ctrl TCCSUP cells. Consistent with this, YBX1 knockdown tumors showed less immunoreactivity of Ki67, a proliferation index (Figure 6B). Western blotting analysis of tissues from these tumors confirmed the knockdown efficiency of YBX1, as well as reduced Myc and HIF1α in sh-YBX1 groups (Figure 6C). Notably, genes involved in glycolysis (Glut1, HK2, PFKL, and LDHA) were also demonstrated to be downregulated in sh-YBX1 groups in comparison to sh-Ctrl group (Figure 6D). By IHC analysis, we noticed that sh-YBX1 knockdown resulted in decreased protein expression of glycolytic enzymes (Glut1, HK2, and LDHA), Myc, and HIF1α *in vivo* (Figure 6E).

## DISCUSSION

Previous studies have shown that YBX1 is a potential prognostic factor in bladder cancer and YBX1 plays tumor-promoting effects in cancer progression [12, 17]. In this study, we for the first time demonstrated the mechanism underlying the malignant roles of YBX1 in bladder cancer.

By analyzing the TCGA bladder cancer gene expression profiles, we identified that genes involved in glycolysis is significantly enriched in high-YBX1 expression group. Consistent with the result from bioinformatic analysis, we confirmed that YBX1 is able to regulate the expression level of glycolytic enzymes, glucose utilization, lactate secretion, ECAR of bladder cancer cells. Previously, no report is available about the regulatory of YBX1 in glucose metabolism. Therefore, our data, as a proof of principle, reveal a novel function of YBX1 in cancer metabolism. And in line with previous report in bladder cancer, knockdown of YBX1 inhibited cell proliferation and promoted cell apoptosis [17]. In contrast to normal cells, cancer cells exhibit dramatic aerobic glycolysis, which sustain the rapid proliferation of cancer cells [6]. We further certified that the prolific and anti-apoptotic effects of YBX1 were largely dependent on glycylosis as 2-DG or lower glucose sufficiently blocked the tumor-promoting effects of YBX1.

It is well-known that Myc and HIF1α are two crucial transcription factors in glycolysis by regulating the expression of several key glycolytic enzymes, including Glut1, HK2, Pfkl, and Ldha [20, 21]. Recently, it has been reported that YBX1 enhances HIF1α protein expression by directly binding to and activating translation of HIF1A messages [8]. Notably, a feed-forward loop between YBX1 and Myc is essential for multiple myeloma cell survival [22]. In this study, by ClueGo analysis, we found that YBX1 participates in a wide variety of DNA/RNA-dependent events. Furthermore, we confirmed that YBX1 is able to regulate the expression of Myc and HIF1α. As both the mRNA and protein of Myc was altered by YBX1, we concluded that YBX1 may regulate Myc expression at transcriptional level at least. Actually, there have no the specific binding sites of YBX1 on Myc promoter. In contrast, Myc can regulate the transcription of the YBX1 promoter. Meanwhile, it has been reported that YBX1 can interact with the RNA-binding protein IGF2BP1 (IGF-II mRNA binding protein 1) stabilizes the Myc RNA by associating with the Coding Region instability Determinant (CRD). Therefore, the regulatory of YBX1 on MYC expression might be mediated its RNA-binding activity rather than the transcription activity. For HIF1α expression, YBX1 was able to influence protein level but not mRNA level, suggesting a translational or post-translational regulatory model. Therefore, further investigations are warranted to fully address this mechanism in bladder cancer.

At last, we determined the potential therapeutic value of targeting YBX1 in bladder cancer. In recent, several *in vivo* loss-of-function studies showed that silencing of YBX1 suppressed tumorigenesis, such as liver cancer [23] and lung cancer [24]. In line with these reports, we found that knockdown of YBX1 resulted in growth inhibition of bladder cancer cells. Specifically, this type of tumor-suppressive of YBX1 loss was associated with decreased glycolytic phenotypes. Suppression of glucose metabolism is a promising targeting avenue to inhibit tumor growth as well as progression. And ample evidence confirmed that targeting glucose metabolism is not only reasonable but also feasible in pre-clinical studies [25, 26]. However, this therapeutic strategy is not satisfactory in clinical trial due to limited efficiency. Alternatively, YBX1 might be an attractive therapeutic approach for bladder cancer.

In conclusion, our findings show that YBX acts as a tumor promoter by targeting Myc and HIF1α to facilitate glycolysis, which is crucial to the malignant phenotypes of bladder cancer cells. Our data highlight the regulatory mechanisms of YBX1 implicated in glycolysis and provide insight into YBX1-based targeted therapy in bladder cancer.

## MATERIALS AND METHODS

### Tissue specimens and animals

The mRNA profiles used in this study were obtained from and The Cancer Genome Atlas (TCGA) database (http://cancergenome.nih.gov). All the animal studies have been approved by the Ethics Committee of QianFoShan Hospital and Shandong University.

### Bioinformatics analysis

Functional pathways related to the oncogenic activities of YBX1 were evaluated using GSEA software. Using the hallmark gene sets deposited in the GSEA MSigDB resource, we identified the differential pathways between the high-YBX1 expression and low-YBX1 expression specimens. For annotation the function of YBX1 positively correlated genes (https://hgserver1.amc.nl/cgi-bin/r2/main.cgi), Cytoscape software and the ClueGo + CluePedia Apps (http://www.cytoscape.org/) were used.

### Cell culture and reagent

The human bladder cancer cell lines J82, TCCSUP, UMUC3, T24, and SW780 were obtained from the Institute of Biochemistry and Cell Biology, Chinese Academy of Science. All cells cultured in DMEM (Dulbecco’s modified Eagle’s medium, Gibco) supplemented with 10% fetal bovine serum and 1% streptomycin-penicillin in a humidified incubator with 5% CO_2_ atmosphere. 2-Deoxy-D-glucose (2-DG) was purchased from Sigma and dissolved in dimethyl sulfoxide (DMSO) to make up a stock solution. CHX and MG132 were purchased from Selleck (Shanghai, China),

### Construction of stably YBX1 knockdown cells

Lentiviral plasmids targeting YBX1 or negative control were designed and synthesized by Genepharma (Shanghai, China). The sequences are shown as follows: sh-1, 5′-CAGGCGAAGGTTCCCACCTTA-3′; sh-2, 5′-CAAGAAGGTCATCGCAACGAA-3′. Combined with the packaging plasmid and envelope plasmid, sh-YBX1 plasmids were transfected into 293 T cell for 48 h to get the lentivirus supernatant. The lentivirus supernatant was then collected and frozen at -80°C for later use. Stable cell lines were established by infecting lentivirus into TCCSUP and SW780 cells and selected with 2 μg/ml puromycin for 2 weeks. And established stable cell lines were maintained in DMEM containing 10% FBS and 0.5 μg/ml puromycin for further experiments.

### Transfection

For overexpression of YBX1 in T24 cells, or overexpression of Myc and HIF1α in TCCSUP and SW780 cells, pcDNA3.1-Myc (#16011) and pcDNA3.1-HIF1α (#18949) plasmids were acquired from Addgene. In brief, the culture medium was replaced by 600 μl of transfection mixture containing 8 μl of Lipofectamine 2000 and 2 μg overexpressing plasmids or empty vector. Transfection was performed using Lipofectamine 2000 (Invitrogen, Grand Island, NY, USA) according to the manufacturer’s instructions. The overexpression efficiency were assessed after 72 h of transfection

### RNA extraction and quantitative real-time polymerase chain reaction (qRT-PCR)

Total RNA from tumor cells was extracted using the Trizol reagent (Ambion). RNA concentration and quality were determined by the ND-1000 spectrophotometer (NanoDrop Technologies). A total of 1 μg total RNA was used to synthesize cDNA using SuperScript® III Reverse Transcriptase (Invitrogen, Life Technologies). The qRT-PCR was performed with 7500 fast (Applied Biosystems) and used to analyze the expression of mRNA in bladder cancer cells. To quantify changes in gene expression, the comparative C_t_ (ΔΔCT method) was used to calculate the relative fold change normalized to β-actin. The PCR primers sequences sequences were listed as follows. YBX1 forward: 5’-CAATGTAAGGAACGGATATGG-3’; reverse:5’-TTCCCCACTCTCACTATTCTG-3’; Glut1 forward: 5’-ATTGGCTCCGGTATCGTCAAC-3’; reverse: 5’-GCTCAGATAGGACATCCAGGGTA-3’; HK2 forward: 5’-TTGACCAGGAGATTGACATGGG-3’; reverse: 5’-CAACCGCATCAGGACCTCA-3’; Pfkl forward: 5’-GCTGGGCGGCACTATCATT-3’; reverse: 5’- TCAGGTGCGAGTAGGTCCG -3’; Ldha forward: 5’- TTGACCTACGTGGCTTGGAAG -3’; reverse: 5’-GGTAACGGAATCGGGCTGAAT-3’; Myc forward: 5’-GTCAAGAGGCGAACACACAAC-3’; reverse: 5’-TTGGACGGACAGGATGTATGC-3’; HIF1α forward: 5’-ATCCATGTGACCATGAGGAAATG-3’; reverse: 5’-TCGGCTAGTTAGGGTACACTTC-3’.

### Western blot analysis

The cells were lysed for protein extraction by a lysis buffer (Beyotime, Shanghai, China). Equal amounts of protein (15 μg per lane) were separated by 10% SDS-PAGE and electrophoretically transferred onto nitrocellulose membranes. The membranes were then blocked with 5% defatted milk. After incubation overnight with primary antibodies against YBX1 (#4202, Cell Signaling Technology, Shanghai, China), Myc (#9402, Cell Signaling Technology, Shanghai, China), HIF1α (ab113642, Abcam, Cambridge, UK), and β-actin (ab8227, Abcam, Cambridge, UK), the second HRP conjugated secondary antibodies were used at room temperature for 1 h. Immunoblots were developed using the enhanced chemiluminescence (ECL) detection system (Millipore). The relative gray scale of the bands was analyzed using Image J software.

### Measurement of glucose and lactate

Glucose levels were determined using a commercial glucose assay kit (Sigma-Aldrich, USA). Glucose uptake was calculated by deducting the detected glucose concentration in the medium from the original glucose concentration. Lactate levels were determined using a lactate assay kit (Biovision, USA) in accordance to the manufacturer’s instruction. All values were normalized on the basis of the BCA protein assay (Thermo Fisher Scientific, USA).

### Measurement of extracellular acidification rate (ECAR)

*In vivo* real-time ECAR was monitored with the Seahorse XF96 Flux Analyser (Seahorse Bioscience) in accordance to the manufacturer’s instructions. Cells at 20,000 per well were seeded in a XF96-well plate containing complete medium. Bladder cancer cells were incubated with unbuffered media followed by a sequential injection of 10 mM glucose, 2 mM (TCCSUP and SW780 cells) or 1 mM (T24 cells) oligomycin and 80 mM 2-DG. All values were normalized on the basis of the BCA protein assay.

### Cell proliferation and cell apoptosis assay

MTT (Sigma-Aldrich, St. Louis, MO, USA) assay was performed to measure cell viability. Briefly, cells with indicated treatment were seeded at 3 × 10^3^ cells per well in 96-well plates. The cell viability was determined at 72 h after YBX1 knockdown or overexpression. Absorbance was recorded at 570 nm using a microplate reader. For cell apoptosis assay, the Caspase-Glo 3/7 assay kit (Promega, USA) in accordance to the manufacturer’s instructions. For Annexin V/PI staining, apoptotic cells were detected using a commercial kit (BD Biosciences). Briefly, 1 × 10^6^ cells were washed twice in cold PBS and resuspended in 1 × binding buffer, followed by addition of 10 μl of annexin V-FITC and 10 μl of propidium iodide into the 100 μl cell suspensions. After incubation for 15 min, cells were analyzed by llow cytometry.

### *In vivo* tumour growth experiment

NOD-SCID BALB/c mice (4-6 weeks old, 16-18g weigh) were managed at SPF Laboratory Animal Center in full accordance with the guidelines by the U.S. National Institutes of Health Guide for the Care and Use of Laboratory Animals. A total of 2 × 10^6^ TCCSUP control or YBX1 knockdown cells were inoculated subcutaneously in the right back of nude mice (n = 5 per group). The growth of the primary tumors was recorded every 4 days. The tumor volume was calculated as V = (width^2^ × length)/2. After mice were sacrificed, tumor specimens were fixed in formalin and embedded in paraffin or directly stored at -80°C. The expression of Ki67 (ab15580, Abcam, Cambridge, UK) was measure by immunohistochemistry. Myc and HIF1α were detected by western blotting. Glycolytic enzymes were analyzed by RT-qPCR.

### Statistical analysis

Data were presented in the form of means ± standard deviation (SD). Group difference was assessed using the unpaired t-test. Correlation between YBX1 and Myc or HIF1α was determined using the Spearman’s test. A two-sided p-value of < 0.05 was considered statistically significant.
